# Honor Those Who Deserve It

**Published:** 1892-10

**Authors:** 


### Editorial.

### HONOR THOSE WHO DESERVE IT.

Original thought is perhaps the rarest of gifts, and the idea has been entertained that nothing in our modern life can be stamped with that quality, all forms being but changes upon the work of past generations of civilization. This is, probably, in its broadest sense true, yet there is unquestionably absolute originality, or else there could be no progress. It is possible that the art of to-day may be a lost art recovered and moulded to suit the ideas of our more advanced life, but it still remains original with this period, and can in no sense be regarded as a legacy in whole from the past.

Who can estimate the amount of loss of original discoveries in the arts and sciences? Who will ever know the number who have died and carried the secret of some valuable addition to the knowledge of the world with them? The answer to these questions can never be given, but every single loss means sooner or later the necessity for a repetition of the same work by others and rediscoveries along all lost lines of work. The world's needs will compel this.

A very singular fact, and one worthy of more attention than is usually given it, is that it is exceedingly rare to find any discovery made by a single individual. The germs of invention will secure a lodgement in several receptive brains at the same period, and the work will proceed independently to one common end. The records of the Patent-Office are full of such cases. What is true of the mechanical side of active life is equally true of problems of thought. To defend originality here is more difficult, and the plagiarist can revert with some degree of plausibility to the assumed fact that all the ideas of to-day are but restatements of those of yesterday, and, therefore, have no rightful owner.

The question, Who can claim originality? has vital importance in all the various relations of active life, but to none more than dentistry.

While the evolution of mentality has had its proportion of loss and gain, and while in the general advance the individual has suffered, it does not follow that this need be continued indefinitely. It is just as important to our conception to historically preserve the originator as the thing originated, yet civilization clings to the latter and forgets the former.

Dentistry can scarcely be said to have a history. Whatever of value it possesses as one of the very important branches of work has accrued in a comparative short term of years, and yet brief as it is the largest proportion of originators are already lost to knowledge.

We have urged the importance of this branch of our subject in a previous article, but the negligence, amounting almost to criminality, still continues with accumulating force, threatening to deprive men not only of the right of invention, but after having freely given their ideas to the profession to have them as freely stolen.

It is time we honored those who deserve honor, and preserve the record for the instruction and emulation of future epochs.

The question naturally follows, To whom should be given honor, the man who scientifically demonstrates a fact and then boldly promulgates it, or he who has discovered the germ and permits it to die? These two classes abound everywhere, and the latter is extremely prolific in dentistry. This is to be expected. The two or three who receive the inspiration of an invention are multiplied to an increasing number from the fact that the incentive to inspiration, necessity, is, and always has been, present here to a greater degree than elsewhere.

There probably never has been an association or convention of dentists where an elaborate scientific statement has been made but that some one would claim precedence of discovery. So common has this been that it has ceased to excite remark. This is wrong, as in our judgment it should be met by a proper investigation and the truth established. For this purpose each society might have a standing committee to whom all such questions should be referred. It must be remembered we are making history, and it is of vital importance that the origin of all things should be established now, and not be left to the uncertainties of a future period.

The two classes of workers before referred to, the one who has scientifically elaborated a subject and given it freely to his colleagues, and the other who has discovered a fact and permitted it to die, have each a place worthy of honorable mention; but he whose work has been based on scientific principles, who has exhausted all available means and in the end has accomplished results, is entitled to the honor of original discovery, and his name should accompany it to remotest generations. On the other hand, the man who has kept his work secret, perhaps not extending it to important conclusions, or not following scientific methods, or, if successful, lacking moral courage to let the world know of his results, has no right of discovery. More than this, he is to be condemned, in that by his want of professional energy or cowardice, or both combined, the profession has been deprived of a valuable accessory to practice.

The truth of these statements has found renewed confirmation recently in two meetings of the profession, one at Niagara Falls, the American Dental Association, and the other at the New York Odontological Society, and both on the same subject,—"The Use of Nitrate of Silver in Dental Caries." At the first the claim was made that this agent had been used forty years ago for a similar purpose. If this be true, and we have no reason to doubt the statement, the literature of the period failed, as far as we are aware, to show it. If any dentist did the work as claimed, how much may not have been lost by the neglect to press the matter upon the profession! When Dr. Stebbins, of Massachusetts, presented this subject after six years of patient investigation, it was regarded as entirely new in the direction in which he had experimented. His work had been thoroughly done, and, having accomplished substantial results, he gave these and the process, leaving it to others to confirm them. In view of these facts, does the credit of this belong to the dead past or the living present?

The remarks of Dr. Brockway in the discussion of this subject before the New York Odontological Society illustrated so admirably a rare class of mind, one with the courage to admit an error, that we cannot omit reproducing them here: "I have been in the habit of using nitrate of silver myself occasionally for many years. I am quite ashamed to mention this fact, inasmuch as I did not have courage enough or did not use it understandingly enough to go on and secure decided results, but I have for the past twenty-five years occasionally touched softened spots, especially on the buccal surfaces of molar teeth, with nitrate of silver with the hope of retarding decay. I have done it almost empirically, and with good results."

Whether Dr. Stebbins's results will be verified or not remains for time to demonstrate; but one thing has been clearly proven,—the necessity of thoroughly doing the work allotted to any one to do, and having left nothing undone to test conclusions, then giving them to the world regardless of consequences.

The profession, then, has its duty, and that is not to permit the discoverer to be buried in oblivion or his labor to be appropriated by others. His reputation is in the hands of his colleagues. He has freely given his work, let them see to it that it is honestly defended.

The time has come for plain words and men with the courage of their convictions.

